# Clinical, Ethical and Financial Implications of Incidental Imaging Findings: Experience from a Phase I Trial in Healthy Elderly Volunteers

**DOI:** 10.1371/journal.pone.0049814

**Published:** 2012-11-16

**Authors:** David J. Pinato, Chara Stavraka, Mark Tanner, Audrey Esson, Eric W. Jacobson, Martin R. Wilkins, Vincenzo Libri

**Affiliations:** 1 The National Institute for Health Research (NIHR)-Wellcome Trust Imperial College Clinical Research Facility, Imperial Centre for Translational and Experimental Medicine, Imperial College, Hammersmith Hospital Campus, London, United Kingdom; 2 Imanova Centre for Imaging Sciences, London, United Kingdom; 3 Sirtris Pharmaceuticals Inc., Cambridge, Massachusetts, United States of America; Cardiff University, United Kingdom

## Abstract

**Background:**

The detection of incidental findings (IF) in magnetic resonance imaging (MRI) studies is common and increases as a function of age. Responsible handling of IF is required, with implications for the conduct of research and the provision of good clinical care.

**Aim:**

To investigate the prevalence and clinical significance of IF in a prospective cohort of healthy elderly volunteers who underwent MRI of the torso as a baseline investigation for a phase I trial. We assessed the follow-up pathway with consequent cost implications and impact on trial outcomes.

**Methods:**

A total of 29 elderly healthy volunteers (mean age 67, range 61–77, 59% female) were eligible at screening and underwent MRI for assessment of visceral and subcutaneous fat.

**Results:**

IF were detected in 19 subjects (66%). Suspected IF of high and low clinical significance were found in 14% and 52% of participants, respectively. Follow up of IF was conducted in 18 individuals, confirming abnormalities in 13 subjects, 3 of whom were recommended for deferred clinical re-evaluation. The remaining 5 subjects had false positive IF based on second line imaging tests. Costs of follow-up medical care were considerable.

**Conclusion:**

MRI abnormalities are common in elderly individuals, as a result of age and non-diagnostic quality of research scans. In the presence of IF in the context of clinical trials, immediate referrals and follow up assessments may be required to rule out suspected pathology prior to exposing trial participants to investigational medicine products (IMP). Unanticipated costs, ethical implication and the possible impact of IF on trial outcomes need to be taken into account when designing and conducting trials with an IMP.

## Introduction

Phase I trials represent a necessary step in the drug development process, in which preliminary data regarding the safety, tolerability, pharmacokinetics and metabolism of novel investigational medicinal products (IMP) are obtained from small groups of healthy volunteers or patients [1].

Alongside traditional pharmacokinetic measures, that indeed represent the primary endpoint of first-time in man (FTIM) studies, initial evidence of pharmacodynamic (PD) effects is increasingly being sought in early phase trials as a secondary study endpoint [2]. While effects on PD endpoints in healthy volunteers may not accurately predict the true clinical outcomes in patients, they may provide initial indication as to whether novel putative therapies are sufficiently promising to justify the conduct of larger-scale longer-term clinical trials [3]. In addition, the use of PD biomarkers in healthy volunteers can facilitate dose selection in future efficacy trials in patients [4].

Imaging studies have gained increasing popularity in clinical research as a reliable, reproducible and minimally invasive source of biomarkers [5]. Different imaging modalities, including magnetic resonance, ultrasound and positron emission tomography (PET), have been variably employed to assess the bioactivity of medicinal compounds across different disease areas, including cardiovascular and metabolic medicine [6], neurosciences [7] and oncology [8].

Among the imaging biomarkers tested in healthy individuals, magnetic resonance imaging (MRI) holds several advantageous properties, including accurate soft tissue contrast features, lack of ionizing radiation and the possibility of estimating physiological parameters, such as blood flow, or to derive human body composition by quantifying the distribution of adipose over muscular tissue [9].

Because of these favorable properties, MRI has been used with increasing frequency in clinical research and, as a result of the increased number of subjects undergoing MRI scans, the detection of incidental findings in otherwise healthy and asymptomatic volunteers has become an ever more common occurrence.

A number of studies conducted in healthy volunteers have reported a considerable proportion of unexpected radiological findings of potential clinical significance following MRI investigation of various anatomical regions, mostly focusing on brain, head & neck districts [10], [11], [12], [13], where the prevalence of findings requiring clinical follow-up or therapeutic intervention ranged from 2 to 10% depending on the examined region.

A more recent study estimated a 13% prevalence of abnormalities of moderate to high significance in whole body MRI scans, reporting a steady increase in the risk of IFs associating with age [14].

The clinical, ethical and emotional implications relating to the detection of IFs represent a renowned challenge in clinical research; most of the implications stemming from the report of radiological abnormalities are inferred from observational studies and may or may not inform an objective cost-benefit analysis for screening of asymptomatic subjects.

Little is known about the impact of IFs in the conduct of phase I trials. This lack of knowledge includes the financial consequences that either sponsors or primary care providers may face from the need of follow-up investigations subsequent to the discovery of unexpected abnormalities.

In this study we report the rate and type of IFs on MRI scans of the torso in a group of healthy elderly volunteers screened for an IMP study and we address the resulting clinical, ethical and financial implications in the specific context of early phase interventional studies.

## Materials and Methods

### Sampled Population

The study population included for the present study is composed of twenty-nine healthy volunteers who were considered for a Phase I single-center study investigating the safety, tolerability, pharmacokinetic and pharmacodynamics properties of a novel compound being developed for the treatment of metabolic disorders (ClinicalTrials.gov reference number NCT00964340). The primary outcomes of the clinical trial are published elsewhere (Libri et al., manuscript submitted).

As part of the screening process, all the volunteers underwent medical history review and full clinical examination. Routine blood and urine tests and an ECG were obtained to confirm subject’s eligibility. The body mass index (BMI) ranged from 18 and 30 kg/m^2^. Subjects with any active medical diagnosis at screening were excluded. Subjects were also ineligible if they had a history of any chronic disease or any clinically significant illness within 3 months of study entry that, in the opinion of the investigators, could risk subject safety or interpretation of the results.

The study was conducted at The National Institute for Health Research (NIHR)-Wellcome Trust Imperial College Clinical Research Facility (formerly known as McMichael-Welcome Trust Clinical Research Facility) within Imperial College London, in compliance with the Declaration of Helsinki and the International Conference on Harmonisation (ICH) and Good Clinical Practice (GCP) guidelines and after formal approval by the competent Research Ethics Committee (reference number 09/H0505/96) and the UK Medicines and Healthcare products Regulatory Agency (MHRA). Written informed consent was obtained from each subject prior to the performance of any study-specific procedures.

### Magnetic Resonance Imaging

Twenty-nine subjects underwent a Magnetic Resonance Imaging (MRI) scan at study baseline to quantify intra-abdominal and subcutaneous adipose tissue. Data were acquired on a Siemens 3T Tim Trio MR scanner (Siemens Healthcare, Erlangen Germany) with the 6 element phased array spine coil and two 4-element phased array flexible torso coils using a 3D volume-interpolated breath-hold examination (VIBE) [15] spoiled gradient echo sequence acquiring two echo times (2.45 ms and 3.675 ms) in order to capture water and fat with and without a 180° phase shift. The imaging volume consisted of up to 5 contiguous axial 3D slabs covering the top of the aortic arch to the femoral heads. Each slab had a field of view of 450mm×267 mm, was acquired in a single 15-second end-expiration breath hold and contained 32 5-mm slices with 1.4×1.4 mm^2^ resolution, using a 7 ms repetition time, 10° flip angle, 6/8 partial Fourier in both phase encoding dimensions and a parallel imaging factor of 2. Complex-valued data from each echo time were decomposed using the Dixon technique into fat-only, water-only, in-phase, and opposed-phase images [16].

### Reporting, Disclosure and Follow-up of Incidental Findings

During consent, every subject was informed about the chance of unexpected findings being discovered during screening tests, in accordance with study patient information sheet, which stated explicitly the following: “there is a possibility that the tests performed during the study will find a medical condition which you did not know about. If this happens your research doctor will discuss the result with you and will, with your permission, refer you to your GP for further medical care”.

Each participant consented to have their MRI scan reviewed by a clinical radiologist to investigate the presence of unexpected findings. A senior consultant radiologist, who interpreted the data independently from the research team, reported the MRI scans.

Clinical correlation of each finding was discussed between senior investigators from the medical team and the reporting radiologist with the aim of facilitating the interpretation of the scans and in order to ensure the provision of the most appropriate follow up plans to the volunteers. Investigators organized follow-up imaging tests after discussion with volunteers according to the principles of good clinical practice.

For the purpose of data analysis, IFs were classified as being of “high” and “low” clinical significance according to their likelihood to have an impact on subject’s health as described previously [14]. Briefly, abnormalities including indeterminate lesions in solid organs and enlarged lymph nodes were classified as being of high clinical significance. Abnormalities of low clinical significance were those who did not require urgent follow up investigation or treatment and included gallstones, diverticulosis and simple kidney or liver cysts.

The estimated costs applying to each individual follow-up examination agreed by the investigators were derived from the local National Health Service (NHS) Trust Imaging Department costing list applying to research studies. The costs of primary care consultations were derived from the Unit Costs of Health and Social Care Report 2009 [17].

### Data Collection and Analysis

Data were collected and analyzed using Microsoft Excel 2010 Version 14.0 (Microsoft Corporation, Washington, USA) implemented with the XLSTAT add-in statistical package.

## Results

Twenty-nine healthy elderly subjects whose clinical characteristics at screening are summarized in **Table 1**, were included in our analysis. The study participants were mostly female (n = 17, 59%) with a mean age of 67.1 years (range 61–77).

**Figure 1:**
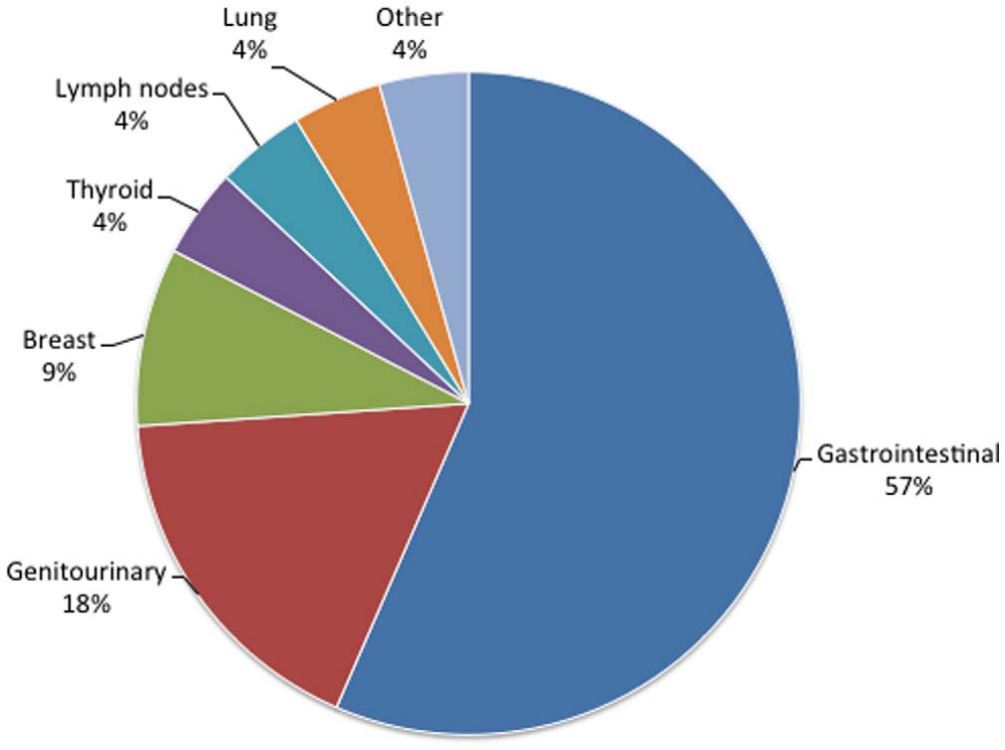
Anatomical site distribution of the incidental findings.

While body MRI scan at baseline revealed one or more abnormalities in 19 subjects (66%), which were considered to be of high and low clinical significance in 4 (14%) and 15 (52%) subjects, respectively. The most commonly affected region was the gastrointestinal tract (57%). The most frequently reported abnormality was the presence of solitary cystic liver lesions in 7 subjects, comprising 30% of all the abnormalities detected. The prevalence of IFs in our prospectively enrolled cohort, categorized by site and degree of clinical significance, are described in **Table 2** and in **Figure 1**.

**Table 1:** Subjects demographics at screening.

Characteristic	N = 29	
Age (years)	Mean (range)	67.1 (61–77)
	SD	±4.1
Sex	Female	17 (59%)
	Male	12 (41%)
Ethnicity	Asian	2 (7%)
	Black	2 (7%)
	White	25 (86%)
BMI (Kg/m^2^)	Mean	25.6
	SD	**±**2.3
Systolic Blood Pressure (mmHg)	Mean	128.1
	SD	±15.3

**Table 2:** The prevalence of incidental findings in elderly healthy volunteers, with categorization of the identified abnormalities by anatomical site and level of clinical significance.

Abnormality	Overall prevalence of incidental findings (n = 29 subjects)	Number of abnormalitiesfound (n = 23)
**High clinical significance**	**4 (14%)**	**8 (35%)**
Multiple liver lesions		2 (9.0%)
Focal liver lesion		1 (4.3%)
Breast lump (single/multiple)		2 (9.0%)
Lung nodules		1 (4.3%)
Mesenteric lymphadenopathy		1 (4.3%)
Thyroid nodule		1 (4.3%)
**Low clinical significance**	**15 (52%)**	**15 (65%)**
Solitary liver cystic lesion		7 (30.0%)
Diverticulosis		2 (9.0%)
Solitary kidney cystic lesion		3 (13.1%)
Prostate enlargement		1 (4.3%)
Gallstones		1 (4.3%)
Rotator cuff cyst		1 (4.3%)
**No abnormalities detected**	**10 (34%)**	

Out of the 19 subjects with at least one MRI abnormality, 18 subjects underwent follow-up imaging investigations within 2 weeks from the referral. The remaining 1 subject did not require follow up investigation as a definitive diagnosis of gallstones was made on the original MRI scan. The most frequently performed diagnostic test was abdominal ultrasound scan (US) in 8 cases.

In 10 of the 18 subjects referred for follow up assessment (56%), IFs were confirmed (e.g. kidney and liver cysts, colon diverticulosis, prostate enlargement) although they were not considered to influence the subject’s health status or trial eligibility and did not require further investigation. In 3 subjects (16%), follow-up scans confirmed an abnormality requiring intervention or long term follow-up: one case of liver cyst with complex echogenic structure and a second subject with multiple thyroid nodules identified on neck US were recommended to have deferred imaging re-evaluation. A third patient with an incidental breast nodule with equivocal mammographic and US features was recommended to have a core biopsy. This patient withdrew consent and was referred to her GP to allow for complete diagnostic characterization and further medical care. After withdrawal of consent, the subject had a core biopsy of her breast lesion performed elsewhere and histopathological examination of the specimen was consistent with fibroadenoma, for which conservative management was recommended. In the remaining 5 individuals (28%) the follow-up scan was negative for the abnormality specified in the MRI scan, confirming a false positive reading in the first instance.

**Figure 2:**
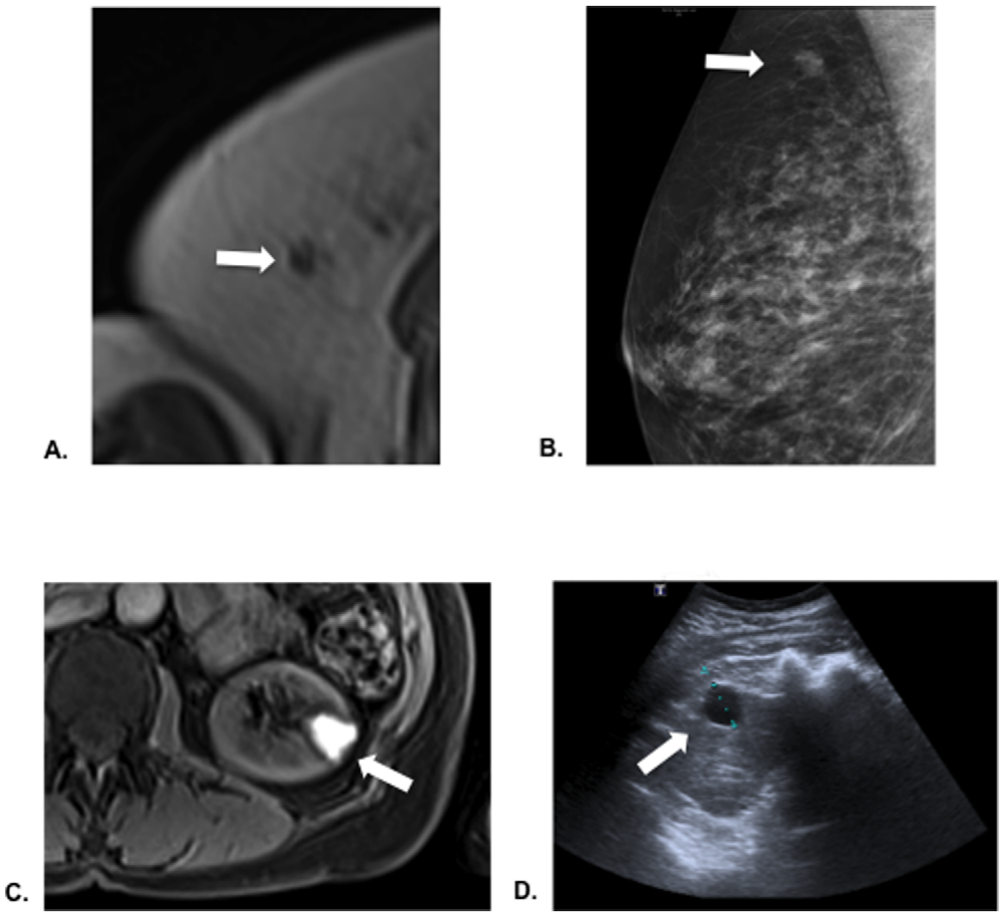
Examples of two subjects with incidental findings from research MRI scans and their associated follow up investigations. **Panel A** shows an in-phase axial Dixon VIBE MR image displaying a lesion (white arrow) in the right breast classified as being of high clinical significance. **Panel B** shows the clinical mammogram image that correlate to **A.** In **Panel C** a cystic lesion arising from the upper pole of the right kidney (white arrow) is seen on a water-only Dixon VIBE MR image and classified as being of low clinical significance. This was further diagnosed as a cortical kidney cyst (Bosniak Type I) on follow up ultrasound scan (**Panel D**).

The diagnostic modality type, costs and clinical outcome emerging from the follow-up investigations arranged for the subjects in whom incidental MRI findings were identified are summarized in **Table 3**. The global costs emerging from the investigation of IFs amount to £ 7,775.00, implying an increase of £ 268.00 in the clinical trial budget per individual patient screened. The mean individual cost for a second-line imaging test was £ 433.00 (95% CI 284–582), ranging from £ 255.00 to £ 1,145.00 depending on the diagnostic modality utilized. The costs generated from the 3 cases for which deferred follow up of IFs was recommended amounted to £ 835.00. Two subjects were referred to their GPs at the end of study for longer-term primary care, whereas the subject with an incidental breast nodule required further in-hospital diagnostic workup as illustrated in **Table 3**. The cost per GP consultation was estimated based on an average 12 minute consultation [17]. Representative images from the original MRI scans and from follow-up radiological investigations are shown in **Figure 2**.

**Table 3:** Summary of the follow up investigations agreed for the diagnostic confirmation of incidental findings: clinical outcomes and emerging costs.

MRI IncidentalFinding	Follow up investigation	Cost (£)	Clinical outcome	Management Plan	Cost (£)
Lung nodules	CT thorax	255.00	NAD/NFA		
Single livercystic lesion	US abdomen	5×345.00	Benign cyst (4 subjects)/NFANo lesion identified (1 subject)		
Multiple liverlesions	US abdomen	2×345.00	Multiple cysts, one with complexfeatures (1 subject)/Deferredre-evaluation suggested	Primary care referral/NFA	36.00
			Fatty infiltration(1 subject)/NFA		
Single liver andkidney cystic lesion	US abdomen & renal tract	2×345.00	Benign cysts (Bosniak type I, 1 subject)/NFA		
			Benign kidney cyst(Bosniak type I), no liverlesion found (1 subject)/NFA		
Single kidneycystic lesion	US renal tract	345.00	Benign cyst (Bosniak type I)/NFA		
Focal liver lesion	US abdomen	345.00	Fatty deposition/NFA		
Breast lump	Bilateral Mammogram& US breast	2×1,145.00	Indeterminate lesion in mammogram,no lesion in US (1 subject)/NFA		
			Likely fibroadenoma (1 subject),biopsy suggested.	Primary care referral	36.00
				Oncology referral	137.00
				US guided biopsy: fibroadenoma confirmed histologically/NFA	590.00
Thyroid nodule	US neck	345.00	Multiple small nodules/Deferredre-evaluation suggested	Primary carereferral/NFA	36.00
Mesentericlymphadenopathy	CT abdomen & pelvis	255.00	Inflammatory changes/NFA		
	**Imaging Costs**	**6,940.00**		**Follow-up Costs**	**835.00**
				**Total Costs**	**7,775.00**

Abbreviations: NAD = No abnormality detected, NFA = No further action recommended.

## Discussion

IFs discovered during research imaging are defined as findings with potential health or reproductive importance whose discovery is unrelated to the purpose and beyond the aims of the study [18].

To date, no general consensus has been reached on how they should be disclosed to subjects or handled [19]. The clear concern is how to pursue further investigations without unduly alarming the participant. The management plan is often devised case by case by the investigators [20].

In the context of interventional studies, however, the implications of IFs are even more complex than in observational research, as even abnormalities with minimal impact on participants’ health may still influence the process of informed consent, recruitment and safety follow-up offered to trial participants. In particular, it is an ethical requirement for trial information sheets to reflect the disclosure policy that researchers would have to adopt if IFs are found [21]. In addition, in the specific area of IMP studies, where individuals may be challenged with potentially toxic new chemical entities, IFs may influence the conduct of the trial, prompting investigators to consider the withdrawal of subjects with abnormalities requiring urgent follow up or whose presence may put participants’ safety at risk if included in the study.

In this paper we report that 66% of the healthy elderly volunteers considered for a Phase I IMP study were found to have IF on baseline MRI scans; a proportion that is twice that previously estimated [14]. This is perhaps not surprising considering the more advanced age of our population compared to previous studies [14]. Nonetheless it is pertinent to the fact that elderly individuals are increasingly recruited into Phase I IMP studies, to fully appreciate age-related variations in the pharmacokinetic profile of the investigational drug under testing [22], or to infer preliminary pharmacokinetic/pharmacodynamic relationships in a subject sample as similar as possible to the foreseen target patient population [23].

It is accepted that the overall prevalence of disease in elderly individuals, even in the absence of clinical symptoms or signs, is higher as a direct consequence of aging [24]. For this reason, the presence of stringent, pre-defined eligibility criteria allows for the detection of clinically significant abnormalities at screening, having the dual aim of avoiding the exposure of subjects with medical conditions to potential harm as well as to protect trial results from confounding factors in assessing the safety of the IMP [25].

Our results show that despite meeting all the entry criteria, 18/29 of the considered subjects demonstrated suspected MRI abnormalities at screening that, in the opinion of the reporting radiologist, required appropriate follow up diagnostic assessment to rule out the possibility that they reflected silent, previously undetected pathologies. The above figures are in line with those described by a larger study of 1192 asymptomatic individuals with a median age of 54 years, in whom 86% of the whole body computer tomography scans reported at least one abnormal finding with further diagnostic evaluation being warranted in 43% [26].

Interestingly, in 72% of the individuals who had a second line diagnostic test, abnormalities were not considered to be of clinical concern in 56% of the subjects, whereas they were of potentially high clinical significance in 16% of the subjects and justified a longer term follow-up including imaging re-evaluation and, in one case, percutaneous biopsy.

The increased sensitivity of whole body MRI in detecting morphological changes with possible pathological significance, in conjunction with the higher prevalence of IF seen in elderly individuals increases the importance of independent review of research scans by senior radiologists in order to optimize the management and follow-up pathway of IF as early as possible [27]. On the other hand, the risk of false positives, which in this study accounted for 28% of the reported lesions, needs to be taken into account when tests of limited diagnostic quality are used(13).

In our study the MRI sequences were designed for delineating anatomical structures and to be acquired within a short breath-hold, using T1-weighted images. Despite being entirely appropriate for the research need, the use of T1-weighted Dixon VIBE sequence had implications with regards to the diagnostic interpretation of the images and did pose some small issues with flow and ghosting artifact in some cases. It is debatable that with the addition of some T2 weighted images, radiological characterization would have been aided and may have reduced the percentage of false positives and therefore the need for second line diagnostic tests.

There are in fact differing views as to whether T2 weighted MRI sequences should be obtained in all subjects or reserved as an additional diagnostic step in those with IF evident on T1 weighted images.

In our study, optimal diagnostic characterization of IF was achieved only after the integrated use of multiple imaging techniques including CT or ultrasound, suggesting that the addition of extra MRI sequences of diagnostic quality may not have facilitated the diagnosis. The characterization of IFs is in fact inherently difficult and poorly reproducible in asymptomatic subjects, suggesting that the T1 MRI technique is only partially contributing to the false positive interpretation rates we found.

The question then arises whether performing additional diagnostic tests that are not specifically meeting the research aims should always be included in study protocols.

Since there is no clear evidence that the follow up of asymptomatic IFs consistently leads to the requirement of referral for further specialist advice and/or the initiation of treatment, the emerging costs arising from the diagnostic procedures involved makes the process of an active search of pathology a point of great controversy [28].

Our data contribute to this controversy by showing that despite implementing an aggressive follow up policy in our trial, no definitive diagnoses of clinical significant pathology could be made in any of the subjects with IFs. However, this conclusion could be drawn at the cost of considerable anxiety to trial participants, which in most cases was unnecessary given the non-clinical significance of IFs in our subjects. As a result, in a context where IFs are highly prevalent but most often not impacting on subjects’ health status, clinical judgment should be used to inform subjects and implement a responsible and cost-effective management plan (13, 27). In such a delicate context, clinicians should pay adequate attention to protect patient’s autonomy by defending their right not to know what was found on research imaging, should this represent the subject’s preference. Regardless of the disclosure policy stated on the trial consent form, sufficient time should be spent during the initial consultation to understand the volunteer’s anticipated preferences in order to facilitate communication and management plans in case IFs are discovered.

In our analysis, we focused our attention on considering the impact of incidental radiological findings on outcomes that are relevant to the conduction of the trial including the eligibility of the screened subjects, dropout rates and the cost implications relating to the follow-up examinations.

In the context of IMP trials, IFs need to be followed up in order to determine their possible clinical significance prior to proceeding with the administration of the IMP. In principle, investigators may be prompted to exclude subjects whose imaging findings cannot be diagnostically characterized, but this would generate a detrimental effect on recruitment and subsequent cost implications to primary care providers. On the other hand, to maintain prospective participants on an IMP trial IFs should be followed up in the interest of subject’s safety and the costs of the required follow-up tests should be covered by the sponsor (as in the present study) and the planned IMP dosing date may be postponed to allow for the completion of the second line diagnostic assessment.

In our experience, the referral process was completed within 1 week from the reporting of the original MRI scan, resulting in a minimal disruption of trial related procedures. However, the fact that this trial was carried out in a dedicated clinical research facility with extensive experience in early phase trials and a direct link to the NHS infrastructures may have contributed to a relative containment of the delays that may not necessarily be replicated in other contexts.

As reported in Table 3, the cost implications stemming from the need to come to a definite diagnostic conclusion were substantial and led to an overall increase in the trial costs. Furthermore, in one case, the fortuitous discovery of a breast nodule requiring further diagnostic characterization induced a participant to withdraw from the study to allow for a more accurate clinical investigation.

The retrospective analysis of a single clinical trial population with a relatively limited sample size should be acknowledged as a potential limitation to the present study. However, the baseline characteristics as well as the eligibility criteria applied to select our prospectively enrolled cohort are those commonly seen in Phase I trials in elderly volunteers [29]. It is conceivable that the policy of our center in determining disclosure and follow-up plan of IFs may not be shared by other research facilities. However, such degree of variability among researchers is acknowledged, since no universally agreed guidelines are in place to guide investigators in the disclosure and management of IFs [30]. It should also be acknowledged that our financial analysis is based on fees applying to the National Health Service in the United Kingdom, which may be dissimilar in other contexts. However, when compared to recently published data, the costs generated by the investigation of IFs we reported do not significantly differ [31], [32].

Despite the acknowledged limitations, our data suggest that the majority of healthy elderly volunteers may be anticipated to have unexpected IFs in MRI scans. In one case the discovery of an IF led to withdrawal of consent. In two other cases long-term follow up was recommended.

The elevated prevalence of abnormalities of low clinical significance and the relatively high rate of false positive readings highlights the importance of accurate reporting of MRI to prevent unnecessary worry of research participants, reduce follow-up costs and optimize management plans. Discrimination between false positives and asymptomatic pathological findings is inherently difficult costly and time-consuming follow-ups. Clinical judgment in harmony with volunteers’ preference should guide follow-up plans to safeguard research participants’ autonomy and ensure correct implementation of ethically approved protocols. The ethical, financial and practical implications originating from our findings should be acknowledged in clinical trial design and management.
